# Development and Validation of a Machine Learning Model to Identify Patients Before Surgery at High Risk for Postoperative Adverse Events

**DOI:** 10.1001/jamanetworkopen.2023.22285

**Published:** 2023-07-07

**Authors:** Aman Mahajan, Stephen Esper, Thien Htay Oo, Jeffery McKibben, Michael Garver, Jamie Artman, Cynthia Klahre, John Ryan, Senthilkumar Sadhasivam, Jennifer Holder-Murray, Oscar C. Marroquin

**Affiliations:** 1 Department of Anesthesiology and Perioperative Medicine, University of Pittsburgh School of Medicine, Pittsburgh, Pennsylvania; 2 Department of Clinical Analytics, University of Pittsburgh Medical Center, Pittsburgh, Pennsylvania; 3 Department of Surgery, University of Pittsburgh Medical Center, Pittsburgh, Pennsylvania; 4 Heart and Vascular Institute, University of Pittsburgh Medical Center, Pittsburgh, Pennsylvania

## Abstract

**Importance:**

Identifying patients at high risk of adverse outcomes prior to surgery may allow for interventions associated with improved postoperative outcomes; however, few tools exist for automated prediction.

**Objective:**

To evaluate the accuracy of an automated machine-learning model in the identification of patients at high risk of adverse outcomes from surgery using only data in the electronic health record.

**Design, Setting, and Participants:**

This prognostic study was conducted among 1 477 561 patients undergoing surgery at 20 community and tertiary care hospitals in the University of Pittsburgh Medical Center (UPMC) health network. The study included 3 phases: (1) building and validating a model on a retrospective population, (2) testing model accuracy on a retrospective population, and (3) validating the model prospectively in clinical care. A gradient-boosted decision tree machine learning method was used for developing a preoperative surgical risk prediction tool. The Shapley additive explanations method was used for model interpretability and further validation. Accuracy was compared between the UPMC model and National Surgical Quality Improvement Program (NSQIP) surgical risk calculator for predicting mortality. Data were analyzed from September through December 2021.

**Exposure:**

Undergoing any type of surgical procedure.

**Main Outcomes and Measures:**

Postoperative mortality and major adverse cardiac and cerebrovascular events (MACCEs) at 30 days were evaluated.

**Results:**

Among 1 477 561 patients included in model development (806 148 females [54.5%; mean [SD] age, 56.8 [17.9] years), 1 016 966 patient encounters were used for training and 254 242 separate encounters were used for testing the model. After deployment in clinical use, another 206 353 patients were prospectively evaluated; an additional 902 patients were selected for comparing the accuracy of the UPMC model and NSQIP tool for predicting mortality. The area under the receiver operating characteristic curve (AUROC) for mortality was 0.972 (95% CI, 0.971-0.973) for the training set and 0.946 (95% CI, 0.943-0.948) for the test set. The AUROC for MACCE and mortality was 0.923 (95% CI, 0.922-0.924) on the training and 0.899 (95% CI, 0.896-0.902) on the test set. In prospective evaluation, the AUROC for mortality was 0.956 (95% CI, 0.953-0.959), sensitivity was 2148 of 2517 patients (85.3%), specificity was 186 286 of 203 836 patients (91.4%), and negative predictive value was 186 286 of 186 655 patients (99.8%). The model outperformed the NSQIP tool as measured by AUROC (0.945 [95% CI, 0.914-0.977] vs 0.897 [95% CI, 0.854-0.941], for a difference of 0.048), specificity (0.87 [95% CI, 0.83-0.89] vs 0.68 [95% CI, 0.65-0.69]), and accuracy (0.85 [95% CI, 0.82-0.87] vs 0.69 [95% CI, 0.66, 0.72]).

**Conclusions and Relevance:**

This study found that an automated machine learning model was accurate in identifying patients undergoing surgery who were at high risk of adverse outcomes using only preoperative variables within the electronic health record, with superior performance compared with the NSQIP calculator. These findings suggest that using this model to identify patients at increased risk of adverse outcomes prior to surgery may allow for individualized perioperative care, which may be associated with improved outcomes.

## Key Points

**Question:**

Can an automated machine learning model accurately identify patients at high risk of adverse outcomes and predict surgical mortality or major complications using only preoperative variables?

**Findings:**

This prognostic study of 1 477 561 patients undergoing surgical procedures found that an automated machine learning model had an area under the receiver operating characteristic curve (AUROC) for mortality of 0.972, 0.946, and 0.956 for the training, test, and prospective set, respectively, and an AUROC for major adverse cardiac and cerebrovascular events or mortality of 0.923 and 0.899 on training and test sets, respectively. The model outperformed the National Surgical Quality Improvement Program Surgical Risk Calculator by 0.048 AUROC.

**Meaning:**

These findings suggest that a machine learning model may be used to optimize perioperative care.

## Teaser

This prognostic study evaluates the accuracy of a machine learning model for identifying patients undergoing surgery who were at high risk of adverse outcomes.

## Introduction

Worldwide, the 2 leading causes of mortality are heart disease and stroke, which combined, account for more than 25% of mortal events (15 million events).^1^ Surprisingly, the third leading contributor to deaths on a global scale is postoperative death within 30 days, estimated to occur among 4.2 million people (7.7%) who die each year worldwide.^1,2^ While it does not constitute its own category in mortality tables published by the Centers for Disease Control and Prevention, the magnitude of 30-day postoperative mortality was approximately the third leading contributor to all-cause death in the United States until COVID-19.^2^ There are an estimated 48.4 million surgical procedures performed in the United States annually,^3^ and 30-day postoperative complications may arise in up to 15% of patients. These were estimated to cost hospitals more than $11 000 per case, amounting to more than $31.35 billion nationally per year.^4,5^ Improving health outcomes after surgery represents an enormous opportunity, and improvement in surgical quality and health care costs is a priority for health care services and payors. Of the 15% of patients who experience complications, 50% are patients considered to be high risk.^4,5^ To reduce surgical complications and improve postoperative outcomes, focus has shifted to preoperative and perioperative care for patients at high risk. However, there are few predictive tools that allow hospitals to identify these individuals with high risk in a timely and accurate manner.

The most popular presurgical tool available to identify patients at high risk is the National Surgical Quality Improvement Program (NSQIP) surgical risk calculator (SRC), a predictive model developed by the American College of Surgeons (ACS) across 393 institutions.^6^ Further analysis of the ACS-SRC has shown a decrease in predictive accuracy locally when applied to unique operations, patients, institutions, and regions. Other institutions have created predictive algorithms, such as the Duke University Institute of Health Innovation’s Pythia calculator, that have outperformed SRC in cross-validation.^4^ The Pythia model evaluates only invasive surgical procedures, does not account for patients with missing data, and necessitates variables that occur during surgery to make a final prediction; thus, it is limited as a preoperative risk model.

The aim of our study was to build, deploy, and evaluate the accuracy flexibility, and applicability of a machine learning model to predict 30-day postoperative mortality and major adverse cardiac and cerebrovascular events (MACCEs) for all patients and procedures exclusively using variables in the patient electronic health record (EHR) prior to the start of surgery. To improve the robustness and accuracy of the model, we used data from a large cohort (>1.25 million patients) to develop and validate this model, conducted further prospective validation on another more than 200 000 unique patients, and deployed the model to make daily predictions for the University of Pittsburgh Medical Center (UPMC) clinical data warehouse (CDW). Finally, we compared the accuracy of our perioperative risk model and NSQIP tool for predicting mortality in randomly selected patients.

## Methods

This prognostic study was approved by the University of Pittsburgh Institutional Review Board (IRB) for a continually rolling surgical cohort using iterative machine learning. The IRB provided a waiver of informed consent because the research presented no more than minimal risk of harm. This study is reported in accordance with the Transparent Reporting of a Multivariable Prediction Model for Individual Prognosis or Diagnosis (TRIPOD) reporting guideline.

### Study Design

The study took place in 3 phases: (1) building and validating a model on a retrospective surgical population, (2) testing the accuracy of the model on a retrospective population, and (3) testing the accuracy of the model on a prospective surgical population. Model predictive performance was measured using the area under the receiver operating characteristic curve (AUROC), sensitivity, and specificity, with a focus on clinical interpretability.

### Setting and Population

The data were obtained on patients who presented for treatment at the UPMC, an integrated delivery and finance health system that includes more than 40 academic and community hospitals, which are interconnected by the same EHRs. All data for this study, which included 20 of the 40 hospitals, are housed in the UPMC Clinical Analytics division CDW, which includes more than 32 million clinical encounters since 2008.

### Data Collection and Outcomes

**Figure 1.**
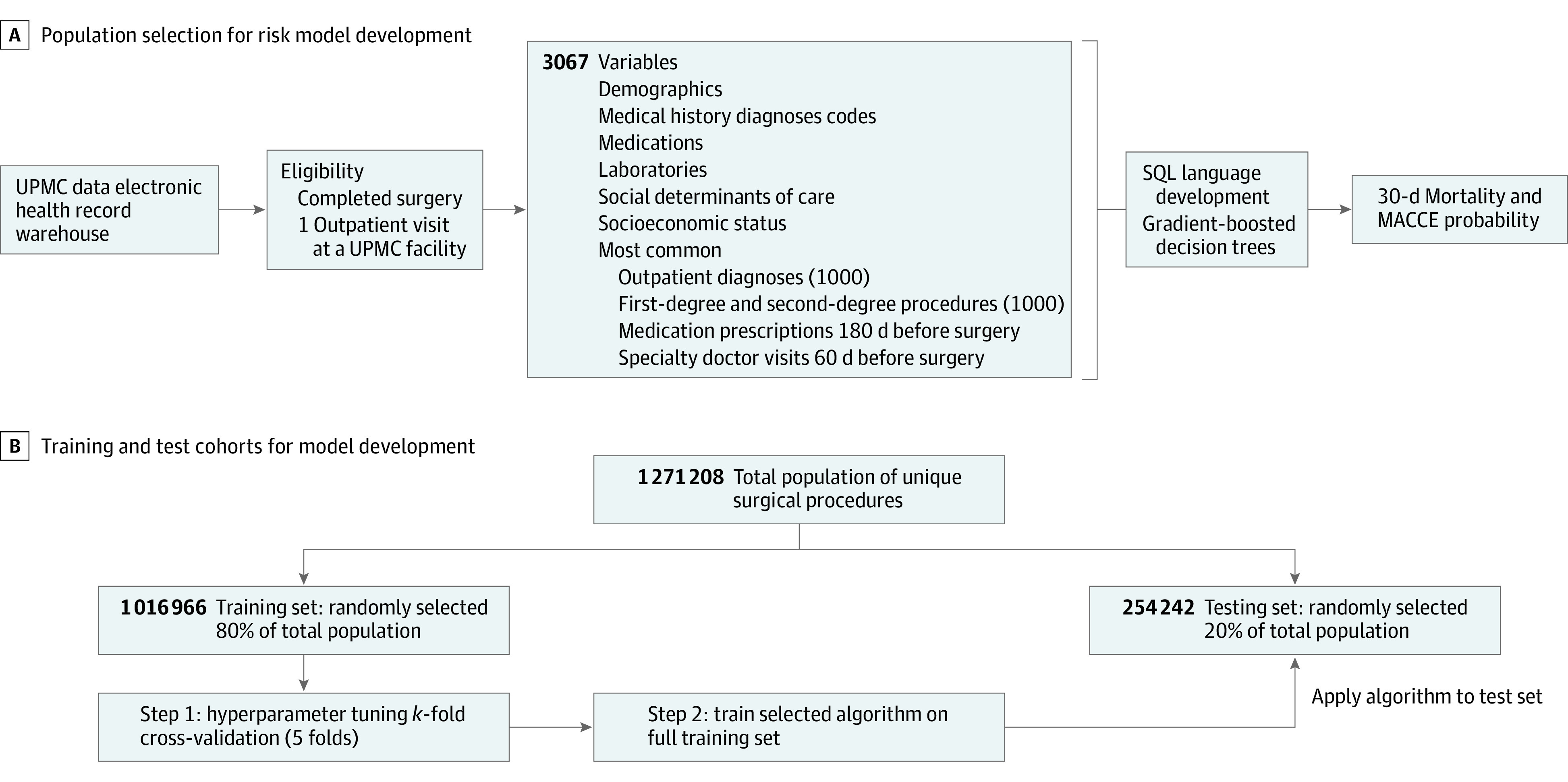
Population Selection and Development of Model A, The population selection for the University of Pittsburgh Medical Center (UPMC) predictive model and input variables for patients undergoing procedures are presented. B, The development schema for the model is presented. The training population consisted of 1 016 966 unique procedures, and the test population included 254 242 unique patients undergoing surgery. MACCE indicates major adverse cardiac and cerebrovascular event; SQL, structured query language.

A cohort of unique surgical procedures using any anesthesiology service (including monitored anesthetic care, and general, local, neuraxial, and regional anesthesia) between December 1, 2012, and May 31, 2019, was identified to develop machine learning models. To be included in the study population, patients must have had a completed surgery and a prior physician visit at a UPMC office (Figure 1A). The final data set for patients undergoing surgery comprised 368 variables with 3067 individual inputs: primary *Current Procedural Terminology* (*CPT*) codes of the scheduled primary surgery, anesthesia type, and patient characteristics, including demographics, historical comorbidities, current medications, most recent laboratory and test values, previous hospitalization information closest to the date of the scheduled surgery, residential socioeconomic factors, and social determinants of care (eTable 1 in Supplement 1). Race and ethnicity (Black, White, or other [including Asian, Latino, Native American, and Pacific Islander]) was identified by recorded information in the EHR. Race and ethncity was included as a patient characteristic given that it may be associated with clinical outcomes. Race and ethncity was listed as Black, White, and other based on information available in the data warehouse. Racial and ethnic groups with smaller sizers were categorized as other due to the sample size and are represented in the study population. We included the 1000 most common diagnoses from office visits 60 days prior to surgery, 1000 most common primary and secondary procedures 1 year prior to surgery, 778 most common pharmaceutical classes of medications prescribed 180 days prior to surgery, and 72 most common specialty physician visits 60 days prior to surgery as independent variables in our model. The most common variables were identified, indexed, stored, and one-hot encoded using the deep learning library Keras version 2.90 (Google). Diagnoses were identified by *International Classification of Diseases, Ninth Revision *(*ICD-9*) and *International Statistical Classification of Diseases and Related Health Problems, Tenth Revision *(*ICD-10*) codes. Previous primary and secondary procedures and the primary procedure for the scheduled surgery were identified by *CPT* codes. Secondary procedures or other unexpected operations performed during the surgery were not included as predictors. The final population was generated using automated structured query language (SQL; joint technical committee of the International Organization for Standardization and International Electrotechnical Commission [ISO/IEC] SC 32 data management and interchange) and Python programming language version 3.9 (Python Software Foundation) code.

The training population and validation cohort for building the model consisted of unique procedures performed between December 2012 and May 2019. To ensure that the model would perform in production and deployment as it did during development and testing, SQL codes were generated to produce these populations, analogous to a deployed state. Subsequently, in the prospective testing phase, the model was silently deployed (clinician blinded) and further evaluated in patients who were to be scheduled for surgery from June 1, 2019, through May 30, 2020, to ensure the accuracy of the model. To avoid data leakage, no unique patient was included in more than 1 data set. The model was then clinically deployed, and prediction of mortality risk was performed and recorded prior to surgery when patients were seen by surgeons or referred to the UPMC Center for Perioperative Care. An application was also developed to allow surgical offices and the UPMC Center for Perioperative Care to have immediate access to risk scores. Using American Heart Association classification, any patient with a risk of 30-day postoperative mortality or MACCE of 5% or greater was said to be high risk.^7^ Finally, we compared the accuracy of the UPMC perioperative risk model and NSQIP tool for predicting mortality in a random selection of approximately 30% of patients (902 of 2901 patients [30.1%]) presenting for surgery at the tertiary care UPMC Presbyterian hospital between April 1 and June 30, 2021. This was also assessed to be the most reasonable in size because of the manual effort required to read through EHRs and manually input information into the risk calculator. Therefore, our team (A.M., S.E., and C.K.) manually entered data from those patients into the NSQIP database calculator. Data were analyzed from September through December 2021.

### Statistical Analysis

#### Model Development

Due to the volume and high dimensionality of clinical data that were collected during clinical care, we used gradient-boosted decision trees as the preferred machine learning method to predict the probability of 30-day postoperative mortality or MACCE. Using American Heart Association criteria, MACCEs were defined as 1 or more of the following recorded in *ICD-10* codes: acute type 1 or type 2 myocardial infarction, cardiogenic shock or acute heart failure, unstable angina, and stroke. Total cardiac death was included in 30-day postoperative mortality, which was attributed to all causes.^8^ While these models are similar to random forests,^9,10^ gradient-boosted decision trees differ in that trees are modeled sequentially by weighting erroneously classified data greater as subsequent models are fit.^11^ They are also able to learn over many iterations and are robust to data missingness and redundant variables. This allows for a more complicated representation across many features to be learned during the training phase of model development. Several state-of-the-art implementations of this type of model exist, including XGBoost (University of Washington), CatBoost (Yandex), and LightGBM (Microsoft). We used LightGBM because of its additional flexibility and training speeds. Our model was built with Python programming language version 3.6 (Python Software Foundation), and we queried our CDW with the pyodbc Python module (Massachusetts Institute of Technology). The level of statistical signficance was *P* < .05.

Model training occurred in an Azure cloud-based Data Science Virtual Machine (Microsoft), equipped with a 2.6-GHz central processing unit, 120 GB of random access memory, and 4 graphics processing units. To prevent overfitting, 5-fold cross-validation was performed during training while tuning 7 hyperparameters: tree depth, number of leaves per tree, learning rate, positive weight scaling, early stopping rounds, L1 regularization, and L2 regularization. Initial hyperparameters were chosen with the Hyperopt library (Python) and then tuned manually. This library fits a tree Parzen estimator^12^ to model the target metric, AUROC, as a function of hyperparameters. This model is used to select hyperparameters in the region of expected highest AUROC for each subsequent trial. This method for initial hyperparametric tuning was chosen instead of random or grid searches, which do not adapt the knowledge of previous tries.^13^ Once hyperparameters were chosen, the model was trained. During training, our model was given 5000 epochs in which to learn while optimizing binary logarithmic loss.^14^

#### Model Interpretability

To help interpret the model, the Shapley additive explanations (SHAP value) method^15^ was used, for which a precedent has been set in the medical field.^16^ A method adopted from the game theory literature for machine learning interpretability, a SHAP value in classification is the mean change in the log odds ratio of the prediction when the feature is added among all other features. Thus, for a given prediction, the SHAP value of a feature is the change in the prediction when the feature is added compared with the baseline. In the past, these values have been extremely computationally intensive. However, a fast algorithm has been discovered to calculate SHAP values for tree-based models, including LightGBM.^15^ SHAP values not only help with model interpretability, but may also serve as further validation of the development of our model. For instance, it would be expected that the most important variables according to SHAP values when fit on the training set should closely resemble the most important variables when the model was fit on the test set. This informed the user that the model was identifying the same predictive patterns in our testing phase as it did during the training phase.

## Results

A cohort of 1 477 561 patients (806 148 females [54.5%]; mean [SD] age, 56.8 [17.9] years; 1 086 286 White [89.8%], 108 813 Black [8.9%], and 14 420 other [1.1%] among 120 9519 patients with race and ethnicity data). with 1 271 208 unique surgical procedures between December 1, 2012, and May 31, 2019, were identified to develop machine learning models. The training population (Figure 1B) consisted of 1 016 966 unique procedures. The model was then validated on a test set of 254 242 unique procedures. Subsequently, when the model was clinically deployed, it was further evaluated prospectively in 206 353 additional patients who were to be scheduled for surgery from June 1, 2019, through May 30, 2020.

**Figure 2.**
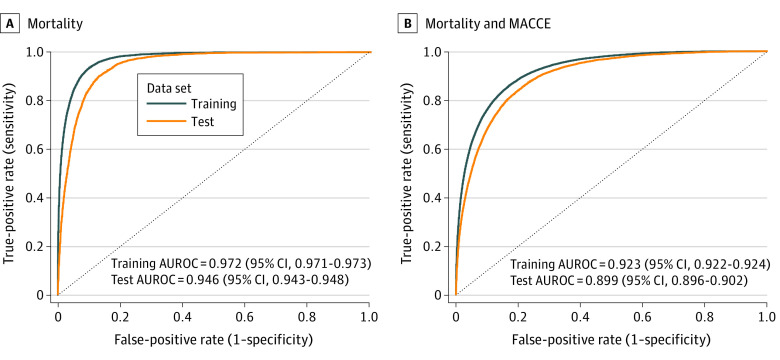
Accuracy of Perioperative Risk Model A, The receiver operating characteristic (ROC) curve is presented for the 30-day mortality model in training and test cohorts. B, The ROC is presented for the 30-day combined mortality and major adverse cardiac and cerebrovascular event (MACCE) model in training and test cohorts.

**Figure 3.**
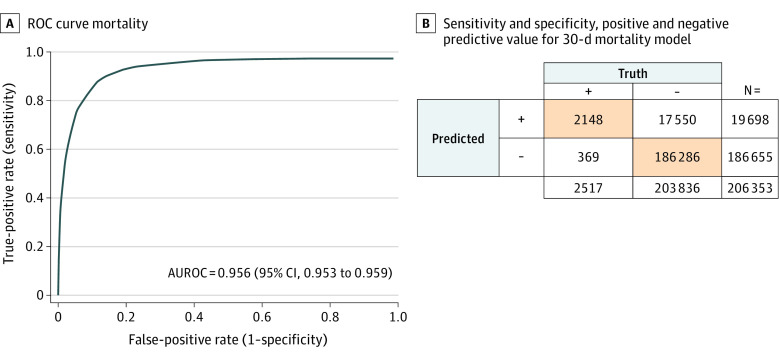
Accuracy of Perioperative Risk Model in Prospective Clinical Implementation The receiver operating characteristic (ROC) curve, sensitivity, specificity, and positive and negative predictive value for the 30-day mortality model in 206 353 prospectively selected patients are presented. AUROC indicates area under the ROC curve.

This study investigated 2 different outcomes: 30-day mortality after surgery and 30-day MACCE or mortality after surgery (Figure 2A and B). The AUROC for mortality was 0.972 (95% CI, 0.971-0.973) for the training set and 0.946 (95% CI, 0.943-0.948) for the test set (Figure 2A). The MACCE or mortality model achieved an AUROC of 0.923 (95% CI, 0.922-0.924) on the training cohort and 0.899 (95% CI, 0.896-0.902) on the test set (Figure 2B). The training and test AUROC for the 2 models were similar and did not suggest overfitting. The prospective evaluation showed an AUROC for mortality of 0.956 (95% CI, 0.953-0.959). Sensitivity was 2148 of 2517 patients (85.3%), and specificity was 186 286 of 203 836 patients (91.4%). The negative predictive value was 186 286 of 186 655 patients (99.8%) (Figure 3).

**Figure 4.**
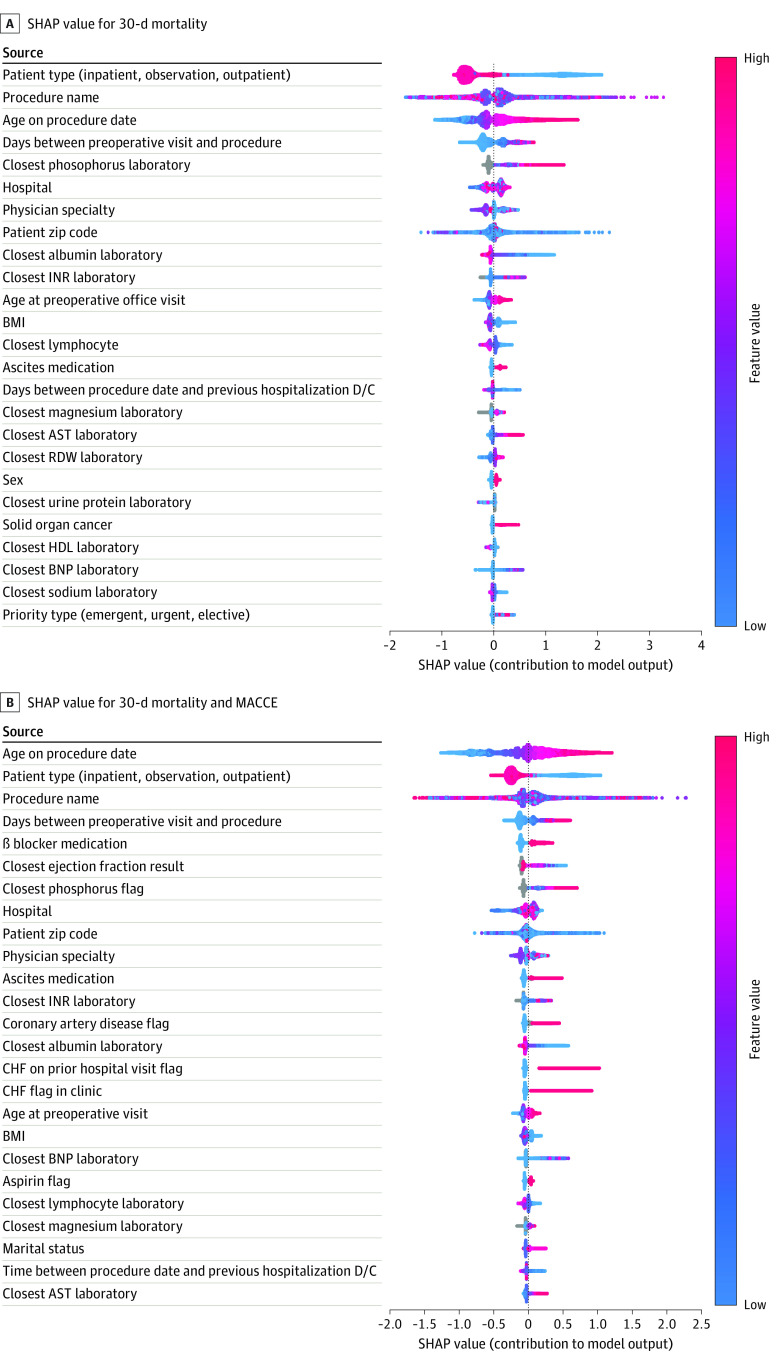
Shapley Additive Explanations (SHAP) Values Identifying Factors Associated With Risk Each variable name is shown on the left-hand side with the variable with the greatest contribution listed at the top. To the right of the variables, there are colored lines, which are individual points that correspond to observations in the population. A higher value for the variable is represented in red, while a lower value for the variable will be shown in blue. A variable with a null value for an observation is visualized in gray. A value farther to the right (ie, a higher SHAP value) indicates that the variable is contributed to a prediction of a positive target, such as mortality or major adverse cardiac and cerebrovascular event (MACCE). AST indicates aspartate aminotransferase; BMI, body mass index (calculated as weight in kilograms divided by height in meters squared); BNP, brain natriuretic peptide; CHF, congestive heart failure; D/C, discharge; HDL, high-density lipoprotein; INR, international normalized ratio; RDW, red cell distribution width.

For model interpretability of assurance, accountability, and actionability, the most important features in the predictive model were reported with SHAP feature attribution values. The top 30 most important features with respect to their log odds of the outcome of interest are reported in Figure 4. SHAP summary plots present the relative importance of the features’ quantitative contribution to each feature and the range and distribution of the feature with respect to model performance.

By SHAP values, age on contact date was associated with the greatest change in output in both models (Figures 4A and 4B). The older the patient undergoing surgery, the more likely they were to have a 30-day MACCE or mortality outcome after surgery. Lower albumin levels (by the most recently measured level) was a significant factor in the mortality model but not the MACCE model. As the value of albumin decreased, it generally contributed to a positive target prediction (ie, a greater increase in mortality). Magnesium (most recent magnesium level) was also associated with a change in outcome in both models. The gray area of the closest magnesium laboratory value corresponds to patients who had null values in this field, which contribute to predictions moving away from a positive target (mortality and MACCE). Binary variables, such as coronary artery disease, were also visualized. In the MACCE or mortality model, patients with coronary artery disease had a higher risk of a 30-day postoperative complication vs those who do not have coronary artery disease. Other variables, such as the scheduled surgery procedure code, were associated with changes in outcomes in both models, but it should be noted that their number values were arbitrary given that this field was hashed to fit the requirements of LightGBM. It is also important to note that these visualizations do not represent a causal model, and thus one should not infer that variable importance by SHAP values were causing or preventing adverse events after surgery.

**Figure 5.**
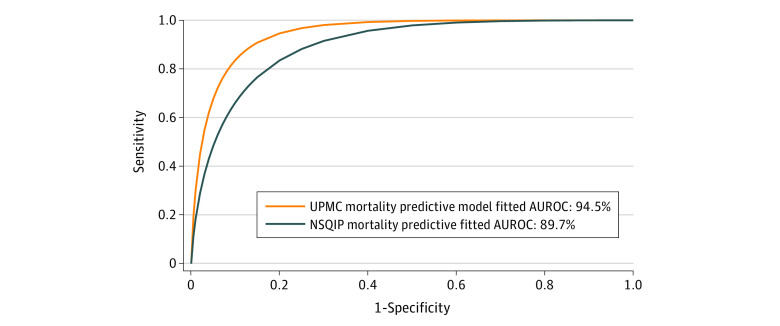
Comparison of Models A comparison of mortality prediction using the University of Pittsburgh Medical Center (UPMC) perioperative risk model and National Surgical Quality Improvement Program (NSQIP) model in 902 patients is presented.

Finally, for comparing the accuracy of risk predictions from NSQIP with those by our UPMC predictive model (Figure 5), 902 patients were included in the analysis. We found that our model outperformed NSQIP as measured by AUROC (0.945 [95% CI: 0.914-0.977] vs 0.897 [95% CI, 0.854-0.941], for a difference of 0.048). The 2 curves were significantly different from one another [*z* = 2.175 [95% CI, 0.005-0.091]; *P* = .03) by Delong test. Specificity was superior in the UPMC model (0.87 [95% CI, 0.83-0.89] vs 0.68 [95% CI, 0.65-0.69]), as were accuracy (0.85 [95% CI, 0.82-0.87] vs 0.69 [95% CI, 0.66-0.72]) and positive predictive value (0.15 [95% CI, 0.10-0.22] vs 0.08 [95% CI, 0.06-0.12]).

After the reliability of our predictive models was validated, the algorithm was deployed so that results could be used in clinical practice. Predictions are made daily at 8:00 am Eastern standard time on every procedure that was scheduled in the UPMC health system the previous day. Prediction scores are reported in the risk prediction application screen (eFigure in Supplement 1). The risk prediction application can also be run on demand for individual patients. The QlikView data analysis application version 12.5 (Qlik) application offers a few advantages when used in practice, the most important of which is that predictions do not have to be made by manually extracting EHR data as is needed in other tools, such as the NSQIP ACS. These scheduled procedures are curated automatically with SQL code. We then fit our developed models onto procedures and write our predictions to the CDW. The data analysis application is then refreshed to absorb the predictions and classify patients based on their risk.

Our model takes into account the scheduled surgical procedure, which is the trigger for the model to run. High- and low-risk thresholds were chosen in our test population to flag the top 10% of patients with the highest risk based on predicted probabilities. eTable 2 in Supplement 1 shows respective thresholds and associated sensitivity, specificity, true and false positives, true and false negatives, and *F*1 scores^17^ on our test population for both models.

## Discussion

In this prognostic study, we developed, validated, and prospectively tested a highly accurate gradient-boosted decision-tree algorithm. The algorithm used information from 1 477 561 patients to predict postoperative 30-day mortality and MACCE using multiple demographic and clinical variables in the EHR within our health system serving a large network of tertiary and community care hospital practices. The new models had high sensitivity and specificity, well beyond the most current, existing predictive models, and used only preoperative data to predict postoperative outcome. We deployed the models to assist in the identification of patients at high risk of adverse outcomes who were scheduled for surgery and, when feasible, preoperative optimization by medical and perioperative teams in the UPMC Center for Perioperative Care.^18^ In many cases, the model has been used not only to evaluate risk and provide optimization through evidence-based guidelines, but also to facilitate a shared decision-making discussion to determine if surgery is truly the best option.^18^

Focusing on preoperative interventions that optimize the health of patients prior to surgery has been shown to be associated with improvement in short- and long-term clinical outcomes.^19,20,21,22^ Effective implementation and scaling of such value-based programs requires automated and accurate risk models that can provide timely information prior to surgery, ideally several weeks before surgery to allow for preoperative interventions to be successful. Further benefits of such risk assessment models may continue in intraoperative and postoperative phases of surgical care with alignment of clinical care teams.

There are other popular surgical risk calculators, of which ACS-SRC is the most widely used risk-assessment tool for helping to predict complications and mortality.^23,24,25,26,27,28^ Thus, we wanted to compare the UPMC automated model to this tool. The ACS-SRC predictive model has shown a decrease in predictive accuracy locally when applied to unique operations, patients, institutions, and regions outside the population in which the ACS-SCR was developed.^23,24,25^ Importantly, ACS requires manual extraction of EHR data for a prediction and cannot accept missing values to provide an output score. Furthermore, no adjustment can be made by adding new variables. In contrast, our algorithms automatically make predictions on a recurring 24-hour cycle, with no manual extraction, based on a refresh of new data entering the EHR. Our models can accept and find patterns in missing data to make a prediction. This makes our tool extremely flexible in its ability to accurately generalize when flagging patients at scheduling who are at high and low risk of adverse surgical outcomes. Our analysis directly comparing 30-day postoperative mortality models from the ACS NSQIP and UPMC predictive model found superior performance of UPMC predictions for our local patients. Many publications have demonstrated the difficulty of the ACS NSQIP calculator to depict postoperative complication risks in many different patient populations,^27,28,29^ although there are very few methods that are superior. The Pythia-based model from Duke University was recently shown to have a better AUROC than the NSQIP in a limited cohort of patients.^4^ Our new risk model builds on the Pythia model with a population many times greater and from varied hospitals, with expanded variables that include clinical, laboratory, imaging markers, and social determinants of health. Notably, this study also found continued superior performance of our model when evaluated prospectively in patients, and it appeared to have superior function to the Pythia-based model (AUROC for mortality, 0.813) and NSQIP.^4^ As our study findings suggest, deploying machine learning models in operations at scale requires EHR embedding and clinical decision support to calculate and trigger workflows.

Additionally, there are other models for the prediction of postoperative MACCE, including the Gupta cardiac risk^30,31^ score and Revised Cardiac Risk Assessment (RCRI).^32,33,34^ The AUROC for Gupta was 0.884 in the final version,^31^ and the RCRI AUROC was 0.806 in the validation cohort.^32^ These 2 models and the ACS-SCR were examined in the prediction of postoperative stroke.^35^ The ACS-SCR score had an AUROC for mortality of 0.836 and 0.833, respectively, and the RCRI had an accuracy of 0.743. By contrast, the UPMC predictive model had an AUROC for mortality of 0.972, which is substantially more accurate than any of the aforementioned scores. The Duke University Pythia model, while more accurate than the ACS-SCR and other existing model, has some limitations. Many patients undergo noninvasive or minimally invasive procedures. This tool cannot be used to accurately flag patients at high risk before the start of surgery. The study was retrospective in its examination of 90 000 prior patients (vs data from >1 450 000 patients in our study with prospective validation). The model does not account for patients with missing data.

### Limitations

There are limitations to our study. While the model was accurate, it was dependent on data in the EHR. If data are absent, the score may not reflect the true risk of the patient. If patients enter our hospital from other systems, after data are entered into the EHR, a proper score cannot be determined until a refresh, which occurs at 12:00 am every day. While many of our patients have comprehensive care at UPMC, there are several cases in which patients do not follow up at UPMC. Although our EHR shares patient medical records with other institutions that have the same agreements, there will be leakage of patients. The actual mortality and MACCE may be higher than suggested in our model because of this leakage. Furthermore, we have not yet validated the model externally on a test set from another institution. This would be a way to further improve the model, which would help avoid data leakage given that it is unlikely the same patient data would be represented in multiple data sets. Our team is making preparations to do this in the near future. A particular concern exists among clinicians that while machine learning may deliver accurate predictive models, the models are largely a black box with little guidance on features or variables that contributed to the risk assignment. Our model used the SHAP value method, with identification of features with the greatest contributions to risk,^15,16^ which is associated with improved interpretability of the predictive risk assessment. SHAP values may also serve as further validation of the development of our model. For instance, we would expect the most important variables according to SHAP values when fit on our training set to closely resemble the most important variables when the model is fit on our test set. This may inform us that the model is identifying the same predictive patterns in our testing phase as it did during our training phase.

## Conclusions

This prognostic study’s findings suggest that perioperative surgical care may benefit from identification of patients based on risk with appropriate resource allocation and care pathways designed to enhance care of patients with complex conditions and at high risk of adverse outcomes. We developed a highly accurate perioperative risk model using only preoperative EHR data that may allow clinicians to treat patients based on their predicted risk of complications or mortality after surgery.

## Notes

Supplement 1. **eFigure.** Risk Prediction Application Screen **eTable 1.** Baseline Characteristics and Model Input Variables for Patients Undergoing Procedure **eTable 2.** Thresholds and Sensitivity, Specificity, True and False Positives and Negatives, and *F*1 Score on Test Population for Both Models

Supplement 2.
Data Sharing Statement
