# Supplementary material for: Development and Validation of a Machine Learning Model to Identify Patients Before Surgery at High Risk for Postoperative Adverse Events
Source: JAMA Netw Open. 2023 Jul 7;6(7):e2322285. doi: 10.1001/jamanetworkopen.2023.22285 (PMC10329211; doi:10.1001/jamanetworkopen.2023.22285)
Supplement: Supplement 1. — eFigure. Risk Prediction Application Screen eTable 1. Baseline Characteristics and Model Input Variables for Patients Undergoing Procedure eTable 2. Thresholds and Sensitivity, Specificity, True and False Positives and Negatives, and F1 Score on Test Population for Both Models [file jamanetwopen-e2322285-s001.pdf]

Supplemental Online Content

Mahajan A, Esper S, Oo TH, et al. Development and validation of a machine learning model to identify patients before surgery at high risk for postoperative adverse events. *JAMA Netw Open*. 2023;6(7):e2322285. doi:10.1001/jamanetworkopen.2023.22285

eFigure. Risk Prediction Application Screen
eTable 1. Baseline Characteristics and Model Input Variables for Patients Undergoing Procedure
eTable 2. Thresholds and Sensitivity, Specificity, True and False Positives and Negatives, and *F*1 Score on Test Population for Both Models

This supplemental material has been provided by the authors to give readers additional information about their work.

© 2023 Mahajan A et al. *JAMA Netw Open.*

eFigure.

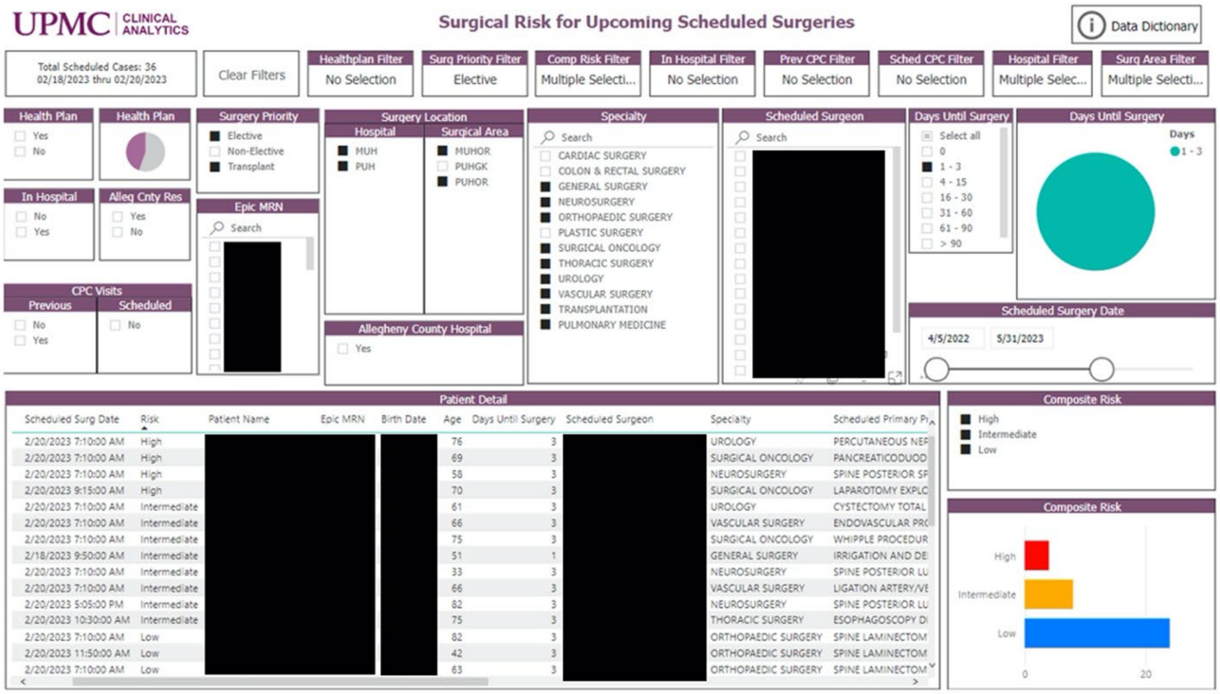

This figure shows the patients upon whom the risk is calculated when the procedure is booked. The output shows the risk score and the bundling of said patients, along with all patient related information and if they were assessed by the CPC. *This enables the surgical and perioperative teams to optimize the high-risk population.*

© 2023 Mahajan A et al. *JAMA Netw Open.*

eTable 1. Prevalence of Patient Characteristics in Study Cohort (of 368 variables evaluated)

**Characteristic**	**Training Set**	**Test Set**	**Validation Set**
	**(N=1,058,791)**	**(N=182,435)**	**(N=201,430)**
Age, mean, median	56.8, 58.9	56.7, 59.6	56.9, 59.8
Female, (No.), %	(593871) 56.1	(101128) 55.4	(111149) 55.2
Race, %			
White	(784992) 89.7	(144126) 90.1	(157168) 90.3
Black	(80874) 9.2	(13491) 8.4	(14448) 8.3
Other	(9588) 1.1	(2332) 1.5	(2500) 1.4
Marital status, %			
Married	(570809) 54.9	(94837) 54.1	(104485) 54.3
Divorced	(91268) 8.8	(14891) 8.5	(16672) 8.7
Single	(262166) 25.2	(49337) 28.1	(54467) 28.3
Other (e.g. widowed, legally separated	(114720) 11.0	(16223) 9.3	(16705) 8.7
BMI, mean, median	29.7, 28.6	30.0, 28.8	30.1, 28.9
Morbid obesity, %	(117288) 13.3	(23290) 14.4	(26846) 15.2
Resting pulse, mean, median	77.1, 76	77.9, 76	78.0, 76
Scheduled specialty, %			
Gastroenterology	(287990) 27.4	(48807) 26.8	(55763) 27.7
Orthopedic surgery	(184342) 17.6	(32241) 17.7	(35072) 17.4
General surgery	(112247) 10.7	(20269) 11.1	(23206) 11.5
Obstetrics/gynecology	(82158) 7.8	(14974) 8.2	(16586) 8.2
Urology	(66597) 6.3	(11626) 6.4	(12767) 6.3
Other	(316514) 30.1	(54001) 29.7	(57681) 28.7
Hospital LOS in past year, %			
0 days	(141873) 32.4	(27636) 37.7	(29871) 36.4
1 day or less	(25152) 5.7	(4259) 5.8	(4538) 5.5
2 days max	(46795) 10.7	(7766) 10.6	(8677) 10.6
5 days max	(85035) 19.4	(13324) 18.2	(14766) 18.0
5+ days	(139363) 31.8	(20296) 27.7	(24162) 29.5
Prior ICU admissions in past year, %			
None	(372271) 85.0	(63874) 87.2	(71032) 86.6
One	(52220) 11.9	(7735) 10.6	(8777) 10.7
Two or more	(13727) 3.1	(1672) 2.3	(2205) 2.7
History of hypertension, %	(334478) 37.9	(59215) 36.6	(65986) 37.4
History of diabetes, %	(141045) 15.9	(243550 15.0	(27937) 15.8
History of atrial fibrillation, %	(48919) 5.5	(8585) 5.3	(9868) 5.6
History of CAD, %	(97732) 11.1	(16861) 10.4	(19043) 10.8
History of stroke, %	(38133) 4.3	(8243) 5.1	(9649) 5.5

© 2023 Mahajan A et al. *JAMA Netw Open.*

History of CHF, %	(49462) 5.6	(89600 5.5	(10448) 5.9
History of pulmonary hypertension, %	(12659) 1.4	(2210) 1.4	(2539) 1.4
History of COPD, %	(82623) 9.4	(17976) 11.1	(20631) 11.7
History of obstructive sleep apnea, %	(92479) 10.4	(17579) 10.8	(20845) 11.8
History of mechanical ventilation, %	(17007) 1.6	(2776) 1.5	(3238) 1.6
History of portal hypertension, %	(51390) 5.8	(9892) 6.1	(11144) 6.3
History of ascites, %	(88414) 10.0	(15044) 9.3	(17689) 10.0
History of cancer, %	(152063) 17.2	(25372) 15.7	(28519) 16.2
History of sepsis, %	(11411) 1.1	(1883) 1.0	(2130) 1.1
Aspirin, %	(273512) 31.0	(48890) 30.3	(52696) 29.9
Beta blockers, %	(225463) 25.6	(39287) 24.3	(43399) 24.6
Diuretics, %	(169508) 19.2	(29448) 18.2	(33079) 18.8
Digoxin, %	(11909) 1.3	(1327) 0.8	(1227) 0.7
Aldosterone blockers, %	(19876) 2.3	(4052) 2.5	(4807) 2.7
Antiplatelet, %	(219981) 24.9	(34890) 21.6	(37299) 21.1
Central antagonist, %	(13559) 1.5	(2318) 1.4	(2678) 1.5
Short-acting bronchodilator, %	(113707) 12.9	(22604) 14.0	(26063) 14.8
Inhaled steroid, %	(56460) 6.4	(10353) 6.4	(11493) 6.5
Insulin med, %	(65065) 7.4	(11855) 7.3	(13576) 7.7
Rapid acting insulin, %	(44705) 5.1	(8080) 5.0	(9011) 5.1
Warfarin, %	(44411) 5.0	(5501) 3.4	(5203)) 3.0
Statin, %	(276954) 31.4	(52928) 32.8	(60502) 34.3
Antidepressant, %	(254456) 28.8	(46695) 28.9	(53196) 30.2
Alanine aminotransferase, mean median	30.3, 22	29.1, 21	28.9, 21
Aspartate aminotransferase, mean median	28.3, 20	27.3, 19	27.0, 19
Bilirubin, mean, median	0.6, 0.5	0.7, 0.5	0.7, 0.5
Albumin, mean, median	3.8, 3.9	3.8, 3.9	3.8, 3.9
Creatinine, mean, median	1.1, 0.9	1.1, 0.9	1.1, 0.9
eGFR, mean, median	61.1, 60	77.9, 76	80.7, 81
RDW, mean, median	14.6, 14.0	14.5, 14.0	14.5, 13.9
Hematocrit, mean, median	39.1, 39.7	39.3, 40.0	39.2, 39.9
Neutrophils, mean, median	68.2, 65.8	69.1, 66.0	69.9, 66.0
Lymphocytes, mean, median	24.6, 24.0	24.6, 23.6	24.5, 23.2
Platelets, mean, median	241, 229	248, 238	253, 241
Glucose, mean, median	113, 100	115, 100	114, 100
Total cholesterol, mean, median	177, 175	175, 173	174, 172
HDL cholesterol, mean, median	50, 48	51, 48	50, 48
Triglycerides mean, median	143, 118	141, 116	138, 115

© 2023 Mahajan A et al. *JAMA Netw Open.*

eTable 2

MORTALITY									
	**Threshold**	**True Positive**	**False Positive**	**True Negative**	**False Negative**	**Accuracy**	**Sensitivity**	**Specificity**	**F1_score**
**0**	0.1	2768.0	25227.0	173137.0	328.0	0.873	0.178	0.894	0.106
**1**	0.2	2441.0	15494.0	182870.0	655.0	0.92	0.232	0.788	0.212
**2**	0.3	2024.0	9660.0	188704.0	1072.0	0.947	0.274	0.654	0.346
**3**	0.4	1534.0	5732.0	192632.0	1562.0	0.964	0.296	0.495	0.505
**4**	0.5	1107.0	3105.0	195259.0	1989.0	0.975	0.303	0.358	0.642
**5**	0.6	681.0	1527.0	196837.0	2415.0	0.98	0.257	0.22	0.78
**MACCE**									
	**Threshold**	**True Positive**	**False Positive**	**True Negative**	**False Negative**	**Accuracy**	**Sensitivity**	**Specificity**	**F1_score**
**0**	0.1	2916.0	54429.0	143935.0	180.0	0.729	0.096	0.942	0.058
**1**	0.2	2596.0	30497.0	167867.0	500.0	0.846	0.143	0.839	0.161
**2**	0.3	2174.0	19474.0	178890.0	922.0	0.899	0.176	0.702	0.298
**3**	0.4	1652.0	12561.0	185803.0	1444.0	0.93	0.191	0.534	0.466
**4**	0.5	1188.0	7978.0	190386.0	1908.0	0.951	0.194	0.384	0.616
**5**	0.6	738.0	5086.0	193278.0	2358.0	0.963	0.165	0.238	0.762

Footnote:

Threshold denotes the probability of the outcome.
The thresholds were defined by clinicians who estimated the “clinically-meaningful” risk to be considered high-, intermediate-, or low-risk. We then saw the observed outcomes at each level of probability in the model and created the risk bins by using the clinically-meaningful estimates that correlated to the probabilities from the model.

F1_score is a machine learning evaluation metric that measures a model’s accuracy. It combines the precision and recall scores of a model using their harmonic mean, and maximizing the F1 score implies simultaneously maximizing both precision and recall.

© 2023 Mahajan A et al. *JAMA Netw Open.*
