# Supplementary material for: Development and Validation of a Machine Learning Model to Identify Patients Before Surgery at High Risk for Postoperative Adverse Events
Source: JAMA Netw Open. 2023 Jul 7;6(7):e2322285. doi: 10.1001/jamanetworkopen.2023.22285 (PMC10329211; doi:10.1001/jamanetworkopen.2023.22285)
Supplement: Supplement 2. — Data Sharing Statement [file jamanetwopen-e2322285-s002.pdf]

Data Sharing Statement

Mahajan. Development and Validation of a Machine Learning Model to Identify Patients Before Surgery at High Risk for Postoperative Adverse Events. *JAMA Netw Open*. Published online July 7, 2023. doi:10.1001/jamanetworkopen.2023.22285

Data
Data available: Yes
Data types: Other (please specify)
Additional Information: Deidentified data requested by the editors **How to access data:** Mahajana@upmc.edu
When available: With publication

Supporting Documents
Document types: None

Additional Information
Who can access the data: researchers whose proposed use of the data has been approved
Types of analyses: For research in this field
Mechanisms of data availability: With signed data access agreement and approval
